# Feline sporotrichosis due to *Sporothrix brasiliensis*: an emerging animal infection in São Paulo, Brazil

**DOI:** 10.1186/s12917-014-0269-5

**Published:** 2014-11-19

**Authors:** Hildebrando Montenegro, Anderson Messias Rodrigues, Maria Adelaide Galvão Dias, Elisabete Aparecida da Silva, Fernanda Bernardi, Zoilo Pires de Camargo

**Affiliations:** Zoonosis Control Center of São Paulo (COVISA/SMS/PMSP), São Paulo, SP Brazil; Federal University of São Paulo (UNIFESP), Department of Microbiology, Immunology and Parasitology, Cell Biology Division, São Paulo, SP Brazil

**Keywords:** Sporotrichosis, Feline, *Sporothrix brasiliensis*, Zoonosis, Emerging infectious diseases, Epidemiology, Cat, *Sporothrix schenckii*, Mycosis, Outbreak

## Abstract

**Background:**

Sporotrichosis is a mycotic infectious disease that is generally acquired by traumatic inoculation of contaminated materials especially from plant debris or through bites and scratches from diseased animals, such as domestic cats. It affects the skin, lymphatic system, and other organs in the warm-blooded host. Etiological agents are embedded in the plant-associated order Ophiostomatales. With essential differences between possible outbreak sources and ecological niche, host-environment interactions are classic determinants of risk factors for disease acquisition. Sporotrichosis outbreaks with zoonotic transmission, such as those that are ongoing in southern and southeastern Brazil, have highlighted the threat of cross-species pathogen transmission. *Sporothrix brasiliensis* has emerged as a human threat owing to the intimate contact pattern between diseased cats and humans in endemic areas.

**Results:**

We describe the recent emergence of feline sporotrichosis in the metropolitan region of São Paulo, Brazil, with an overwhelming occurrence of *S. brasiliensis* as the etiological agent. A phylogenetic and a haplotype approach were used to investigate the origin of this epidemic and the impact of feline transmission on genetic diversity. During the last 3-year period, 163 cases of feline sporotrichosis were reported in São Paulo with proven *S. brasiliensis* culture. The haplotype diversity of feline *S. brasiliensis* isolates revealed the expansion of a clonal population with low genetic diversity. Haplotype analysis confirmed that isolates from São Paulo shared the haplotype originated in the long-lasting outbreak of cat-transmitted sporotrichosis in Rio de Janeiro, which differed from the haplotype circulating in the Rio Grande do Sul epidemic.

**Conclusions:**

The fast spread of sporotrichosis in a short period of time highlights the potential for outbreaks and suggests that the mycosis may affect an urban population with a high concentration of susceptible felines. The feline sporotrichosis epidemic shows no signs of slowing, and this epidemiological pattern may require specific public health strategies to control future outbreaks.

**Electronic supplementary material:**

The online version of this article (doi:10.1186/s12917-014-0269-5) contains supplementary material, which is available to authorized users.

## Background

Epidemics caused by new and old fungal agents have emerged and re-emerged over time as a threat to the health of vertebrate hosts [1]. The great global burden of fungal infections in animals is specially observed as a result of a pathogen-host shift or a recent introduction of a pathogen in a susceptible host population [2–4]. Domestic animals are at risk of developing several mycotic diseases that can be directly transmitted to humans; however, such diseases are often neglected by health systems. Because domestic animals have intimate contact with their owners, they play an important role in the emergence of human infections; this situation is also common in the developing world, where environmental conditions are juxtaposed with inadequate public health infrastructure. Reducing the public health risks from zoonosis outbreaks in urban areas requires different prevention and control strategies, as their increased frequency during recent decades may be related to poverty, poor sanitation, and anthropogenic changes in the environment.

Sporotrichosis is a neglected disease of humans and animals. The disease occurs worldwide in the form of sapronoses and zoonoses, mainly in tropical and subtropical regions [4–7], and is the most frequent subcutaneous mycosis in Latin America [8]. Since it was first noted in the United States in 1898, this mycosis has been described as a disease of occupational risk, affecting farmers, gardeners, and agricultural workers. However, recent epidemics have demonstrated the potential for zoonotic transmission of the disease, and have nearly always involved cats as the main source of infection [9,10]. Around the world, the classical type of transmission relies on the traumatic inoculation of contaminated plant material in the environment. In contrast, in the alternative type of transmission, bites and scratches from a diseased cat effectively disseminate the fungus. Either route of infection begins by affecting the skin locally with the development of a nodular ulcerated lesion, and eventually spreads out from the site of trauma through the lymphatic system and causes damage to other organs of the warm-blooded host [11,12]. Zoonotic sporotrichosis is highly frequent in the southern [4,5,13] and southeastern [4,5,14,15] regions of Brazil, and animals usually experience the severe form of sporotrichosis.

Traditionally, sporotrichosis has been attributed to the dimorphic fungus *Sporothrix schenckii sensu lato* (*s.l.*). Multigene phylogenies have clarified species boundaries within cryptic isolates [16] and led to the proposal of the *S. schenckii* complex, which comprises a clinically important clade that includes *S. brasiliensis* (clade I), *S. schenckii sensu stricto* (*s. str.*) (clade II), *S. globosa* (clade III), and *S. luriei* (clade VI) [17,18]. Host susceptibility, species distribution, and sensitivity profile to antifungal agents are all divergent among closely related species [4,5,19,20]. A high prevalence of *S. brasiliensis*, the most virulent species in the complex [20,21] and geographically restricted to Brazil [4,5,7,22], has been reported in cats [4]. Rodrigues *et al.* [4] suggests that the thermal resistance exhibited by *S. brasiliensis* may be an important mechanism of adaptation to the feline body, and may partially explain the success of *S. brasiliensis* infection over the remaining species in the complex. Indeed, the cat-cat contact pattern during fights and the cat-human contact pattern of scratches and bites may also support the success of horizontal disease transmission in a short period of time [4,5], because the fungus does not die with the feline, and can be transmitted to the next warm-blooded host. The increased proximity between cats and humans favors the emergence of sporotrichosis in Brazil.

Since the 1990’s, the epidemiological profile of sporotrichosis has changed from a low-prevalence disease to a major health problem that affects people living in neglected urban areas [4,5]. Its prevalence may reach epidemic proportions over time. In the metropolitan area of Rio de Janeiro, sporotrichosis is estimated to account for more than 3,800 feline, 4,000 human, and 120 canine cases in the period from 1998 to 2012 [23–25]. Massive zoonotic transmission has also been detected in the southern region of Brazil [5,13,26], with characteristics similar to the ongoing epidemic in Rio de Janeiro.

In contrast to the major ongoing epidemics in other provinces of Brazil, during the past 20 years São Paulo state has reported a basal number of sporotrichosis cases, nearly always unrelated to feline transmission types [5,27]. The Zoonosis Control Center of São Paulo (ZCC-SP) has performed an epidemiological surveillance service among feral cats since 2008. In December 2010, a few cases of sporotrichosis in cats were reported to our service; since then, an increasing number of feline cases have been identified in São Paulo and in two of its neighboring cities. Here, we report the molecular epidemiology of *Sporothrix* species as an emerging pathogen among felines in the metropolitan area of São Paulo and discuss its relevance in one of the most populous regions of the Americas.

## Results

**Figure 1:**
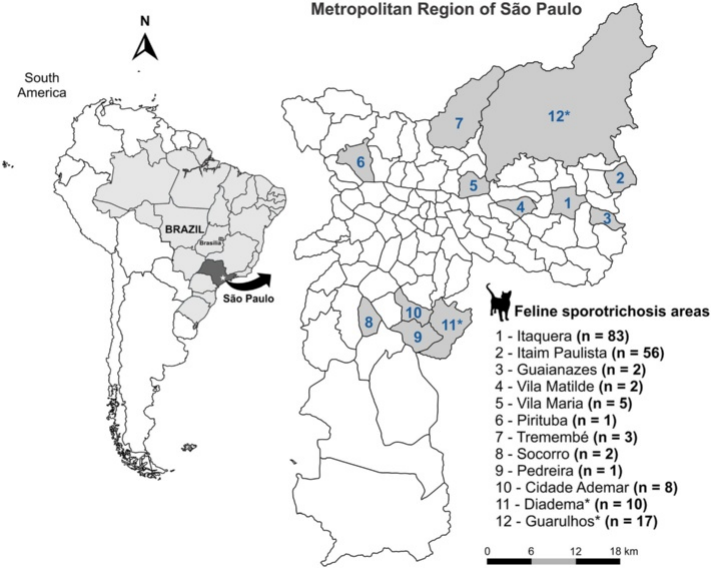
**Spatial distribution of feline sporotrichosis in the metropolitan region of São Paulo.** The most affected area is Itaquera, in eastern São Paulo, where early outbreaks were detected in 2011. *The cities of Guarulhos and Diadema also reported cases of feline sporotrichosis.

**Figure 2:**
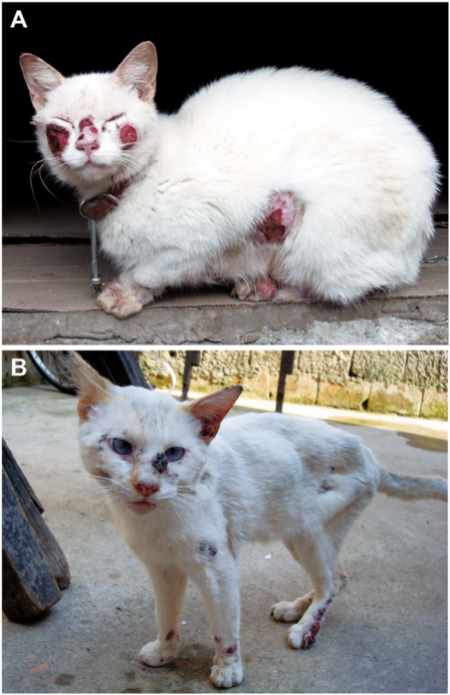
**Clinical aspects of feline sporotrichosis. (A)** Wet, ulcerated skin lesions, often particularly concentrated in the cephalic region. **(B)** Weight loss during the evolution of the disease.

**Figure 3:**
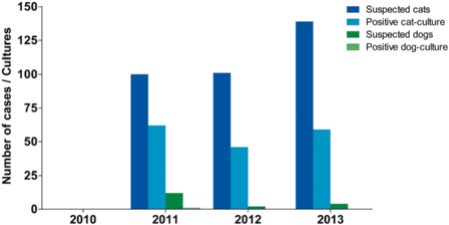
**Temporal evolution of the feline sporotrichosis epidemic in the metropolitan region of São Paulo.** The constant number of positive cats indicates the maintenance of cat-transmitted sporotrichosis.

The first suspected cases of feline sporotrichosis emerged in March 2011 in the region of Itaquera, an urban area with a high population density. Cases are ongoing in the most neglected areas, which have limited access to basic sanitation and public health services (Figure 1). One hundred sixty-three out of 279 clinical samples from cats (58%) and 1 out of 11 samples from dogs (8%) were positive for several *Sporothrix* spp. in the city of São Paulo. Figure 2 shows the clinical aspects of feline sporotrichosis. In the metropolitan area of São Paulo, in the cities of Diadema and Guarulhos, 10 (100%) and 17 of 40 (43%) feline clinical samples were positive for *Sporothrix*, respectively (Table 1). Judging from the number of diseased animals with proven cultures for several *Sporothrix* spp., the sporotrichosis epizootic has shown no signs of slowing during a 3-year period (2011-2013) (Figure 3).

**Table 1:** **Sporotrichosis in feline and canine samples collected from different cities of São Paulo State, Brazil (March 2011 to April 2014)**

**City**	**District**	**Host**	**Positive**	**Negative**	**Positivity**
**São Paulo**	Cidade Ademar	Feline	8	4	67%
	Guaianazes	Feline	2	0	100%
	Itaim Paulista	Canine	0	2	0%
		Feline	56	23	71%
	Itaquera	Canine	1	9	10%
		Feline	83	80	51%
	Pedreira	Feline	1	1	50%
	Pirituba	Feline	1	0	100%
	Socorro	Feline	2	3	40%
	Tremembé	Feline	3	0	100%
	Vila Maria	Feline	5	3	63%
	Vila Matilde	Feline	2	2	50%
**Subtotal**		Canine	1	11	8%
		Feline	163	116	58%
**Diadema**		Canine	0	1	0%
		Feline	10	0	100%
**Guarulhos**		Canine	0	3	0%
		Feline	17	23	43%
**Total**		Canine	1	15	6%
		Feline	190	139	58%

Typical *Sporothrix* colonies were grown from the liver and spleen of animal #1, while fragments from lung and gut were negative. In animal #2, only fragments from the gut were positive for *Sporothrix*. Molecular analysis revealed that the etiological agent was *S. brasiliensis*. Cultures of several *Sporothrix* spp. were obtained from fecal samples from both necropsied cats (M1727/11 and M1732/11). A single environmental sample of feces collected in a sand heap was also positive for *S. brasiliensis* (M039/12).

Positivity for environmental samples was low. All 17 samples from sand piles and a soil sample were negative for *Sporothrix* spp. out of five samples of bark from trees with signs of cat scratches, only one (M1753/11) was positive for *Sporothrix* species. A sample (M1239/11) of decaying wood from a tree branch where a feline with sporotrichosis climbed and fell was also positive for *Sporothrix*. Unfortunately, we were unable to provide a pure culture because of the constant growth of contaminants; therefore, we were unable to identify the exact *Sporothrix* species.

**Figure 4:**
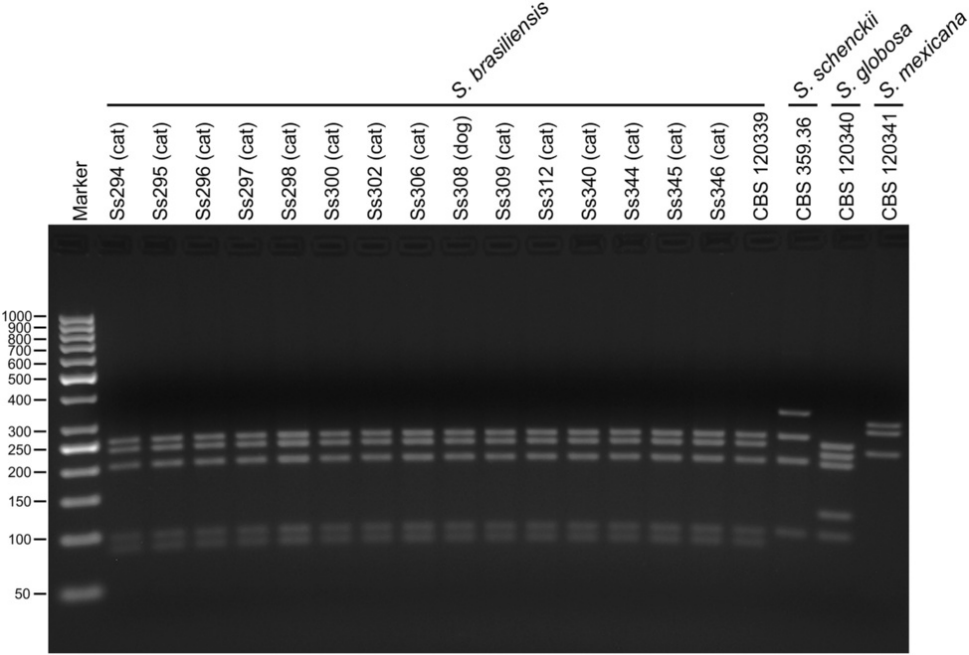
**Genotyping of feline sporotrichosis isolates by PCR-RFLP.** Representative profiles of 16 samples are shown. Positive controls: *Sporothrix brasiliensis* (CBS 120339), *S. schenckii* (CBS 359.36), *S. globosa* (CBS 120340). The amplicons were sized by comparison with bands of known size in the 100-bp DNA Step Ladder (Promega).

Restriction fragment length polymorphism patterns were obtained from *Sporothrix* isolates (Additional file 1) after enzymatic digestion of the *CAL* products with *Hha*I enzyme. Fragments were 251, 232, 198, 96, and 85 bp in length, which is compatible with the restriction profile of *S. brasiliensis* (CBS 120339). A representative gel containing 16 clinical samples is shown in Figure 4.

**Figure 5:**
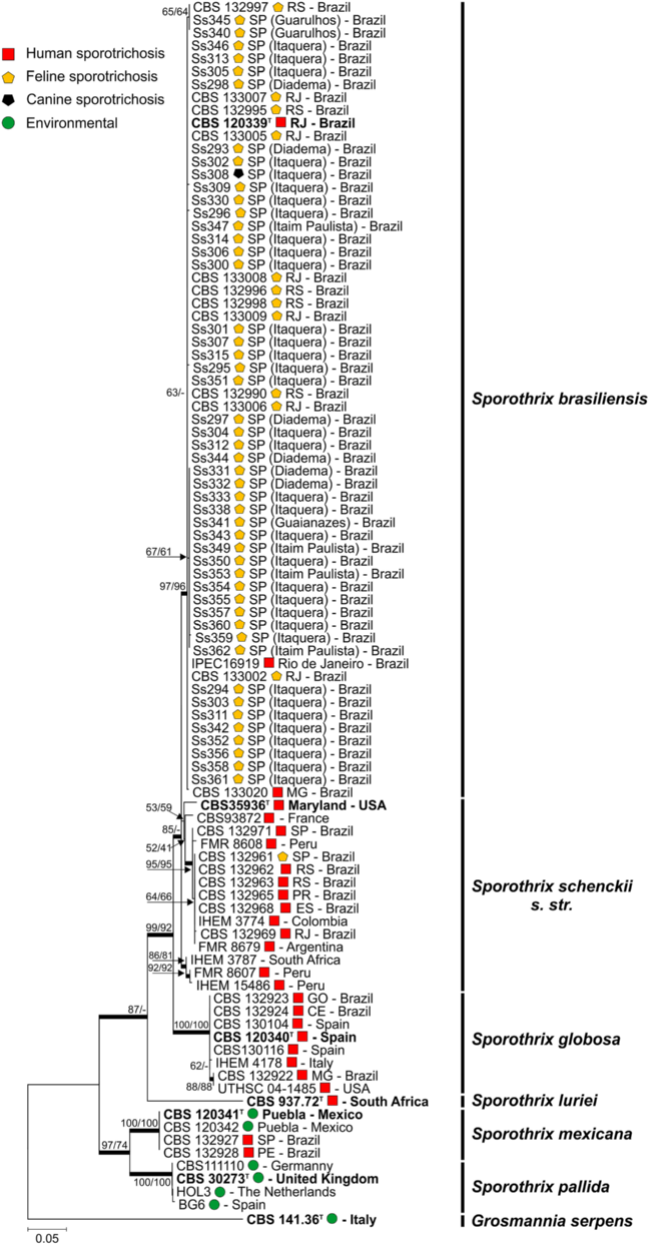
**Phylogenetic analysis using the maximum likelihood method based on sequences from the calmodulin-encoding gene.** The percentage of replicate trees in which the associated taxa clustered together in the bootstrap test (1000 replicates) is shown next to the branches (NJ/ML). The evolutionary distances were computed using the Tamura 3-parameter method (T92 + I). All positions containing gaps and missing data were eliminated. Further information about isolate source and GenBank accession number can be found in the Additional file 1.

A single *CAL* amplicon of approximately 800-900 bp was observed for all isolates. The complete alignment included 95 sequences (47 generated in this study and 48 retrieved from previous investigations). Aligned sequences of *CAL* were 729 bp long, including 355 invariable characters, 216 variable parsimony-informative (29.6%), and 133 singletons. A phylogenetic tree was constructed using Maximum likelihood (model T92 + I) with 1,000 bootstrap replications (Figure 5). The 95 operational taxonomic units were distributed into 7 main groups, 6 of which had been detected in previous studies [4,5,22]. We used the fungus *Grosmannia serpens* (CBS 141.36) as an outgroup [28]. Phylogenetic analyses of the 47 evaluated *Sporothrix* spp. revealed that they all belonged to the species *S. brasiliensis*, and that all were closely related to the type strain CBS 120339.

After speciation, the haplotype diversity of feline *S. brasiliensis* isolates was assessed regarding the *EF1-α* dataset (see Additional file 1). We compared the sequences generated from the recently isolated samples from São Paulo to the ongoing epidemics in Rio de Janeiro and Rio Grande do Sul. The haplotype number for *EF1-α* was low (3 haplotypes: H9, H11 and H12) [4]. Haplotype analysis revealed that isolates from São Paulo shared the same haplotype (H9) from the Rio de Janeiro epidemic (see Additional file 2), which differed from the haplotype circulating in the Rio Grande do Sul epidemic (H11 and H12).

## Discussion

We used molecular tools to investigate the sudden emergence of feline sporotrichosis caused by *S. brasiliensis* in the metropolitan region of São Paulo, the most populated city in Brazil. Sporotrichosis is the most important subcutaneous mycosis that affects animals [4,5,9,10]. Infections caused by *S. brasiliensis* are remarkable among the genus *Sporothrix* because of their intense pathogenicity to the vertebrate host [20,21]. Thus far, *S. brasiliensis* is geographically restricted to Brazil [4,5,22]. To the best of our knowledge, this is the first report of a cat-transmitted epizootic in this area.

Since the identification of the first cases of animal sporotrichosis in São Paulo 3 years ago (2011), there has been a predominance of cases in the southeastern area, specifically in Itaquera and Itaim Paulista districts, denoting an epidemic character (Figure 1). Based on this initial outbreak, the ZCC-SP conducted active searches in households, which resulted in finding other cases of feline sporotrichosis. To date, a total of 83 feline cases and one canine case have been identified in Itaquera, and 56 feline cases have been recorded in Itaim Paulista (Figure 3). As new active searches are conducted in São Paulo, new cases are detected, suggesting that the epidemic is unlikely to end spontaneously. It is important to note that a small number of cats with sporotrichosis have also been found in other districts of the city, as well as in other cities of the same metropolitan area of São Paulo, such as Diadema (10 feline cases) and Guarulhos (17 feline cases) (Table 1; Figure 1). These findings indicate the spread of the epidemic, and lead us to believe that the transmission of this disease might have a silent character.

Molecular data for 48 animals revealed that the outbreak is caused by *S. brasiliensis*. In this study, we have successfully identified the isolates of feline sporotrichosis by *CAL*-RFLP, as suggested by Rodrigues et al. [29]. The use of *CAL*-RFLP considerably reduced the cost of molecular identification in this epidemic scenario. The overwhelming prevalence of *S. brasiliensis* during outbreaks is in agreement with results obtained by Rodrigues et al. [4] regarding feline-transmitted epidemics in Rio de Janeiro and Rio Grande do Sul. In addition, low genetic diversity was observed during the early phase of this epidemic, in agreement with previous results [4,5]. A haplotype network based on *EF1-α* suggests that the predominant haplotype among felines in São Paulo and the haplotype observed in Rio de Janeiro are the same. Although this information may not be conclusive because of the low number of markers used, it still indicates that the disease is spreading from Rio de Janeiro. The state of Rio de Janeiro is bordered by Minas Gerais, Espírito Santo, and São Paulo, and their geographic proximity may support this finding. With particular differences in intensity and frequency of cases, the occurrence of feline sporotrichosis has also been recorded in Minas Gerais [4,5] and Espírito Santo [30,31].

The prevalence of *S. brasiliensis* in cats, but not dogs, in the same geographic area is remarkable. This epidemic profile observed in São Paulo was also detected earlier in Rio de Janeiro and Rio Grande do Sul [5,13–15,32–34]. The success of *S. brasiliensis* epidemics must consider a complexity pathogen-host-environment interplay, including: (a) high susceptibility of the feline host and high virulence of the pathogen; (b) feline habits; and (c) recent introduction of *S. brasiliensis* in a susceptible urban population of felines. Animal sporotrichosis was first described in São Paulo in 1907 by Lutz and Splendore in naturally infected rats [35]. Freitas *et al.* [36,37] reported a series of feline and canine cases in São Paulo, however, we cannot conclude that *S. brasiliensis* was the species involved since Rodrigues *et al.* [4,5] reported that *S. schenckii s. str.* may also infect cats, however with a significantly lower frequency.

The role of cats in the fungal transmission is a key factor in understanding the evolution of disease transmission and emergence in urban areas. This may require the development of specific surveillance programs and control measures by the appropriate authorities. Characteristics of cats’ behavior, particularly fighting during copulation, territorial dispute, or intimate contact, lead to deep scratches and bites, which enable traumatic inoculation of the fungus. The possibility of transmission (and, consequently, transmission to humans) is intensified in areas where non-sterilized animals roam freely, resulting in intimate contact. In Itaquera region, where the majority of the sporotrichosis cases were identified, dogs and cats usually have free access to the street, and most of them are not sterilized.

In the present study, it was possible to isolate *S. brasiliensis* from the organs of necropsied animals at ZCC-SP, is in agreement with previous reports from Schubach et al. [38], who described the isolation of *S. schenckii s.l.* from tissue samples from lung, liver, spleen, lymph nodes, heart, and kidney taken postmortem from ten cats with sporotrichosis. *Sporothrix brasiliensis* were also isolated from feces collected from the small intestine of both necropsied cats, as well as from feces collected from a pile of sand in Itaquera. These findings introduce new insights regarding the ecology of *S. brasiliensis*. Feces from diseased cats may contaminate the soil, creating an environmental reservoir for *S. brasiliensis* and becoming a new source of contamination for animals or humans. Furthermore, cats habitually bury their feces in sand or soil and sharpen their claws on tree bark, enabling the initial contamination of the claw by the fungus. Cats have retractable claws and the fungus may be retained superficially in the animal’s body [9,10]. Moreover, cats’ habit of cleaning themselves by licking can lead to contamination of the oral mucosa, which renders biting and scratching effective for deep implantation of the fungus in cutaneous and subcutaneous sites on other animals and humans. Another possibility that may render soil a source of contamination is the inappropriate disposal of carcasses of animals that died with sporotrichosis, such as backyard burial, or even throwing the animals into the wastelands [4,33].

*Sporothrix* was also isolated from a sample of decaying wood and from the bark of a tree, showing that the fungus is present in the environment within the studied transmission area. However, the rate of positivity among environmental samples was low, which may be related to the sampling strategies, seasonality, and number of evaluated samples. Failure to isolate pathogenic *Sporothrix* spp. embedded in the *S. schenckii* complex from the original source of infection in the environment is not unusual [39–41].

## Conclusions

The recent introduction of *S. brasiliensis* to the metropolitan area of São Paulo has resulted in the permanent infection of felines during the 3 years since it was first detected. We observed striking similarities between the São Paulo epidemic and the long-lasting outbreak of cat-transmitted sporotrichosis and those observed in Rio de Janeiro and Rio Grande do Sul. Moreover, it is unlikely that the epidemic remains limited to cats. The threat of cross-species pathogen transmission can lead to the risk of a massive epidemic for humans in these areas [5] and poses a significant challenge for public health systems. Strategies to control the spreading of the disease may include the education of population about the main aspects of *Sporothrix* transmission, animal sterilization programs, treatment, and prophylaxis, as well as development of campaigns to avoid abandonment of diseased animals by their owners in the most affected areas.

## Methods

### Animal and clinical samples

From March 2011 until April 2014, we studied a total of 345 animals from São Paulo city and neighboring cities that were suspected of having sporotrichosis. Pet owners were informed about the risk of zoonotic transmission of sporotrichosis, and verbal informed consent was obtained by a professional from the ZCC-SP, before the collection of the samples. Suspected animals had apparent cutaneous lesions throughout the body, especially in the cephalic region. The lesions were usually wet, with secretion, but were dry in rare cases. Clinical samples were collected from wet lesions with sterile swabs and sent to the laboratory on the same day. However, if it was not possible to send the samples to the laboratory on the day of collection, they were placed in transport media (Stuart Media) and refrigerated at 4°C. Dry lesions were scraped, and the crusts were collected in sterile flasks. A professional from the ZCC-SP collected all samples. This study was performed in accordance with guidelines for good laboratory practice, and every effort was made to minimize suffering. Ethical approval was provided by the Institutional Committee (Universidade Federal de São Paulo 0244/11).

### *Sporothrix* spp. *isolation and identification*

Clinical samples were directly inoculated on Mycosel Agar slants (Becton Dickinson, Sparks, MD, USA) in duplicate and incubated at 25°C for 30 days. Suspected colonies were subcultured on Sabouraud dextrose agar plates (Becton Dickinson, Sparks, MD, USA). Macroscopic and microscopic characteristics were applied to the dichotomous key to clinical species of the *S. schenckii* complex [18].

### Organs

Two cats that presented with terminal disseminated sporotrichosis were subject to gross necropsy under aseptic conditions after euthanasia at ZCC-SP. The cats’ organs (liver, lung, spleen, and gut) were removed aseptically, cut into small fragments, and macerated in 2 mL of sterile saline solution; 0.5 mL were plated in Mycosel agar plates (in duplicate) and incubated at 25°C for 30 days. Gut samples were well rinsed with sterile saline solution before sample processing. The suspected colonies were isolated and identified as described above.

### Fecal samples

Fecal samples from necropsied cats were collected from the gut. In addition, two environmental feces samples were collected from a sand heap in the Itaquera region, in the backyard of a residence where diseased cats lived. Each fecal sample was diluted in sterile saline solution (1:10 dilution) with chloramphenicol (200 mg/L), vigorously homogenized for 5 min, and allowed to settle for 15 min. Samples of the supernatant (0.5 mL) were plated on Mycosel agar plates (Becton Dickinson, Sparks, MD, USA) in duplicate and incubated at 25°C for 30 days. The suspected colonies were isolated and identified as described above.

### Environmental samples

Environmental samples (n =24) were collected in order to identify potential reservoirs and sources of contamination in neighboring Itaquera, where most of the cases of feline sporotrichosis were found. Sand samples (n =17) were collected from sand piles that contained cat feces; a soil sample (n =1) was collected from a square to which the feral cats had common access at night; bark samples (n =5) were collected from trees with signs of feline scratches near a known home of diseased cats; a sample of decaying wood (n =1) was collected for mycological investigation. Samples were processed as described above, inoculated on Mycosel agar plates (in duplicate), and incubated at 25°C for 30 days.

### Molecular characterization

*Sporothrix* colonies were grown on potato dextrose agar slants (Becton Dickinson, Sparks, MD, USA) for 10 days at 25°C. DNA was extracted and purified from fungal colonies by following the Fast DNA kit protocol (MP Biomedicals, Vista, CA, USA) [22]. The calmodulin (*CAL*) locus region was amplified directly from genomic DNA by polymerase chain reaction (PCR) using the degenerated primers CL1 (5′-GAR TWC AAG GAG GCC TTC TC-3′) and CL2A (5′-TTT TTG CAT CAT GAG TTG GAC-3′) [42], which amplified an 800-bp amplicon corresponding to exons 3 through 5. The *CAL* sequence was used for taxonomy purposes. The translation elongation factor-1 alpha (*EF1-α*) locus region was amplified and sequenced using the primers EF1-F (5′-CTG AGG CTC GTT ACC AGG AG-3′) and EF1-R (5′-CGA CTT GAT GAC ACC GAC AG-3′), as described by Rodrigues et al. [4]. We used *EF1-α* information to compare the ongoing epidemics in Rio de Janeiro, Rio Grande do Sul, and São Paulo. Only PCR products that produced single bands were sequenced. Amplified products were gel purified with the Wizard® SV Gel and PCR Clean-Up System (Promega, Madison, WI, USA), following the manufacturer’s instructions. PCR products were sequenced directly in two reactions with forward and reverse primers to increase the quality of the sequence data (*Phred* >30).

The sequencing reactions were conducted using the BigDye® Terminator v3.1 Cycle Sequencing Kit (Applied Biosystems, Inc., Foster City, CA, USA) and the sequencing products were determined using an ABI 3730 DNA Analyzer 48-well capillary sequencer (Applied Biosystems, Inc., Foster City, CA, USA). Sequences generated in both senses were assembled into single sequences via CAP3 implemented in BioEdit software [43]. Sequences were aligned with MAFFT version 7 [44], and retrieved alignments were manually edited to avoid mis-paired bases. Sequences were exported as FASTA files for BLAST search at http://www.ncbi.nlm.nih.gov/BLAST. All sequences were deposited online at GenBank (Additional file 1).

### Phylogenetic reconstructions

Relationships among *Sporothrix* isolates collected during the São Paulo outbreak were determined by phylogenetic analysis of *CAL* sequences and comparison to reference strains (see Additional file 1) [4,5,16–18,22,45]. Maximum Likelihood and Neighbor-joining methods were employed to complete phylogenetic analyses using MEGA6 [46]. Considering the Bayesian information criterion (BIC) and Akaike information criterion (AIC) [47], the Tamura 3-parameter model (T92 model) [48] was found to be the best evolutionary model for the *CAL* sequence. The model was applied assuming that a certain fraction of sites are evolutionarily invariable. Trees were estimated using 1,000 bootstrap replicates [49]; gaps and missing data were not included in the analysis.

### Haplotype network

Haplotype analysis based on *EF1-α* sequences (see Additional file 1) were estimated using DnaSP software version 5.10 [50] in order to visualize differences and diversity among *S. brasiliensis* isolates recovered from zoonotic outbreaks in São Paulo, Rio de Janeiro, and Rio Grande do Sul [4]. Gaps and missing data were excluded from the calculations. Median-joining networks [51] were obtained and visualized using Network 4.610 software (Fluxus Technology).

### *CAL* restriction fragment length polymorphism

Molecular characterization was also performed by polymerase chain reaction–restriction fragment length polymorphism (PCR-RFLP) as an alternative molecular approach. The partial *CAL* gene was amplified using the primers CL1 and CL2A [42] as described above and digested with *Hha*I as described elsewhere [29]. Digested products were electrophoresed on 2.5% (w/v) agarose gels for 90 min at 100 V in the presence of GelRed^TM^ (Biotium, Hayward, CA, USA). We included a lane loaded with 100-bp DNA Step Ladder (Promega, Madison, WI, USA), as well as one positive control from each of the following reference strains: *S. brasiliensis* (CBS 120339), *S. schenckii* (CBS 359.36), *S. globosa* (CBS 120340), and *S. mexicana* (CBS 120341). The bands were visualized using the L-Pix Touch (Loccus Biotecnologia, São Paulo, Brazil) imaging system under UV illumination.

### Availability of supporting data

All the supporting information is included as additional files.

## Additional files

Additional file 1:
**Strains, species, origin, CAL and EF1-α, and GenBank accession numbers of** ***Sporothrix*** **spp. isolates used in this study.** All sequences were deposited online at GenBank (http://www.ncbi.nlm.nih.gov/genbank). Additional file 2:
**Median-joining haplotype network of** ***Sporothrix schenckii*** **complex isolates, comparing all** ***EF1-α*** **haplotypes described in the ongoing epidemics in Rio de Janeiro, Rio Grande do Sul, and São Paulo.** Isolates recovered in the São Paulo epidemics (2011-2013) share the same haplotype (H9) as previous outbreaks in Rio de Janeiro (1998-2012) reported by Rodrigues et al. [4]. The size of the circumference is proportional to the haplotype frequency. Isolates are coded, and their frequencies are represented by geographic region of isolation. Black dots (median vectors) represent unsampled or extinct haplotypes in the population.

## Abbreviations

*s.l.*: *sensu lato*
*s. str.*: *sensu stricto*
ZCC-SP: Zoonosis Control Center of São Paulo
*CAL*: Calmodulin
*EF1-α*: Translation elongation factor-1 alpha
ML: Maximum likelihood
NJ: Neighbor-joining
BIC: Bayesian information criterion
AIC: Akaike information criterion
T92: Tamura 3-parameter method
PCR-RFLP: Polymerase chain reaction–restriction fragment length polymorphism
