# Supplementary material for: Feline sporotrichosis due to Sporothrix brasiliensis: an emerging animal infection in São Paulo, Brazil
Source: BMC Vet Res. 2014 Nov 19;10:269. doi: 10.1186/s12917-014-0269-5 (PMC4244058; doi:10.1186/s12917-014-0269-5)
Supplement: Additional file 1: — Strains, species, origin, CAL and EF1-α, and GenBank accession numbers of Sporothrix spp. isolates used in this study. All sequences were deposited online at GenBank (http://www.ncbi.nlm.nih.gov/genbank). [file 12917_2014_269_MOESM1_ESM.doc]

**Additional file 1.** Strains, species, origin, *CAL* and *EF1-α*, and GenBank accession numbers of *Sporothrix* spp. isolates used in this study. All sequences were deposited online at GenBank (http://www.ncbi.nlm.nih.gov/genbank).

| **Isolate code** | **CBS Number** | **Species** | **Source** | **Origin** | **Genbank code** | | **References** |
| --- | --- | --- | --- | --- | --- | --- | --- |
|  |  |  |  |  | ***CAL*^a^** | ***EF1-α*^b^** |  |
| Ss54 | CBS 132990 | *Sporothrix brasiliensis* | Feline sporotrichosis | Rio Grande – RS, Brazil | JQ041903 | KC576569 | [1-3] |
| Ss152 | CBS 132995 | *Sporothrix brasiliensis* | Feline sporotrichosis | Pelotas – RS, Brazil | KC693865 | KC576596 | [2, 3] |
| Ss153 | CBS 132996 | *Sporothrix brasiliensis* | Feline sporotrichosis | Pelotas – RS, Brazil | KC693866 | KC576597 | [2, 3] |
| Ss156 | CBS 132997 | *Sporothrix brasiliensis* | Feline sporotrichosis | Pelotas – RS, Brazil | KC693869 | KC576600 | [2, 3] |
| Ss157 | CBS 132998 | *Sporothrix brasiliensis* | Feline sporotrichosis | Pelotas – RS, Brazil | KC693870 | KC576601 | [2, 3] |
| IPEC16490^T^ | CBS 120339 | *Sporothrix brasiliensis* | Human sporotrichosis | Rio de Janeiro – RJ, Brazil | AM116899 | KC576606 | [4, 5] |
| IPEC16919 | - | *Sporothrix brasiliensis* | Human sporotrichosis | Rio de Janeiro – RJ, Brazil | AM116898 | KC576607 | [4, 5] |
| Ss245 | CBS 133005 | *Sporothrix brasiliensis* | Feline sporotrichosis | Rio de Janeiro – RJ, Brazil | KC693878 | KC576619 | [2, 3] |
| Ss246 | CBS 133002 | *Sporothrix brasiliensis* | Feline sporotrichosis | Rio de Janeiro – RJ, Brazil | KC693879 | KC576620 | [2, 3] |
| Ss247 | CBS 133006 | *Sporothrix brasiliensis* | Feline sporotrichosis | Rio de Janeiro – RJ, Brazil | KC693880 | KC576621 | [2, 3] |
| Ss248 | CBS 133007 | *Sporothrix brasiliensis* | Feline sporotrichosis | Rio de Janeiro – RJ, Brazil | KC693881 | KC576622 | [2, 3] |
| Ss249 | CBS 133008 | *Sporothrix brasiliensis* | Feline sporotrichosis | Rio de Janeiro – RJ, Brazil | KC693882 | KC576623 | [2, 3] |
| Ss250 | CBS 133009 | *Sporothrix brasiliensis* | Feline sporotrichosis | Rio de Janeiro – RJ, Brazil | KC693883 | KC576624 | [2, 3] |
| Ss265 | CBS 133020 | *Sporothrix brasiliensis* | Human sporotrichosis | Uberlândia – MG, Brazil | JN204360 | KC576636 | [3, 6] |
| Ss293 | - | *Sporothrix brasiliensis* | Feline sporotrichosis | São Paulo - Diadema – SP, Brazil | KJ002394 | **-** | This study |
| Ss294 | - | *Sporothrix brasiliensis* | Feline sporotrichosis | São Paulo - Itaquera – SP, Brazil | KJ002395 | **-** | This study |
| Ss295 | - | *Sporothrix brasiliensis* | Feline sporotrichosis | São Paulo - Itaquera – SP, Brazil | KJ002396 | **-** | This study |
| Ss296 | - | *Sporothrix brasiliensis* | Feline sporotrichosis | São Paulo - Itaquera – SP, Brazil | KJ002397 | **-** | This study |
| **Isolate code** | **CBS Number** | **Species** | **Source** | **Origin** | **Genbank code** | | **References** |
|  |  |  |  |  | ***CAL*^a^** | ***EF1-α*^b^** |  |
| Ss297 | - | *Sporothrix brasiliensis* | Feline sporotrichosis | São Paulo - Diadema – SP, Brazil | KJ002398 | **-** | This study |
| Ss298 | - | *Sporothrix brasiliensis* | Feline sporotrichosis | São Paulo - Diadema – SP, Brazil | KJ002399 | **-** | This study |
| Ss300 | - | *Sporothrix brasiliensis* | Feline sporotrichosis | São Paulo - Itaquera – SP, Brazil | KJ002400 | **-** | This study |
| Ss301 | - | *Sporothrix brasiliensis* | Feline sporotrichosis | São Paulo - Itaquera – SP, Brazil | KJ002401 | **-** | This study |
| Ss302 | - | *Sporothrix brasiliensis* | Feline sporotrichosis | São Paulo - Itaquera – SP, Brazil | KJ002402 | **-** | This study |
| Ss303 | - | *Sporothrix brasiliensis* | Feline sporotrichosis | São Paulo - Itaquera – SP, Brazil | KJ002403 | **-** | This study |
| Ss304 | - | *Sporothrix brasiliensis* | Feline sporotrichosis | São Paulo - Itaquera – SP, Brazil | KJ002404 | **-** | This study |
| Ss305 | - | *Sporothrix brasiliensis* | Feline sporotrichosis | São Paulo - Itaquera – SP, Brazil | KJ002405 | **-** | This study |
| Ss306 | - | *Sporothrix brasiliensis* | Feline sporotrichosis | São Paulo - Itaquera – SP, Brazil | KJ002406 | **-** | This study |
| Ss307 | - | *Sporothrix brasiliensis* | Feline sporotrichosis | São Paulo - Itaquera – SP, Brazil | KJ002407 | **-** | This study |
| Ss308 | - | *Sporothrix brasiliensis* | Canine sporotrichosis | São Paulo - Itaquera – SP, Brazil | KJ002408 | **-** | This study |
| Ss309 | - | *Sporothrix brasiliensis* | Feline sporotrichosis | São Paulo - Itaquera – SP, Brazil | KJ002409 | **-** | This study |
| Ss311 | - | *Sporothrix brasiliensis* | Feline sporotrichosis | São Paulo - Itaquera – SP, Brazil | KJ002410 | **-** | This study |
| Ss312 | - | *Sporothrix brasiliensis* | Feline sporotrichosis | São Paulo - Itaquera – SP, Brazil | KJ002411 | **-** | This study |
| Ss313 | - | *Sporothrix brasiliensis* | Feline sporotrichosis | São Paulo - Itaquera – SP, Brazil | KJ002412 | **-** | This study |
| Ss314 | - | *Sporothrix brasiliensis* | Feline sporotrichosis | São Paulo - Itaquera – SP, Brazil | KJ002413 | **-** | This study |
| Ss315 | - | *Sporothrix brasiliensis* | Feline sporotrichosis | São Paulo - Itaquera – SP, Brazil | KJ002414 | **-** | This study |
| Ss330 | - | *Sporothrix brasiliensis* | Feline sporotrichosis | São Paulo – SP, Brazil | KJ002415 | **-** | This study |
| Ss331 | - | *Sporothrix brasiliensis* | Feline sporotrichosis | Diadema – SP, Brazil | KJ002416 | KJ002373 | This study |
| Ss332 | - | *Sporothrix brasiliensis* | Feline sporotrichosis | Diadema – SP, Brazil | KJ002417 | KJ002374 | This study |
| **Isolate code** | **CBS Number** | **Species** | **Source** | **Origin** | **Genbank code** | | **References** |
|  |  |  |  |  | ***CAL*^a^** | ***EF1-α*^b^** |  |
| Ss333 | - | *Sporothrix brasiliensis* | Feline sporotrichosis | São Paulo – SP, Brazil | KJ002418 | KJ002375 | This study |
| Ss338 | - | *Sporothrix brasiliensis* | Feline sporotrichosis | São Paulo – SP, Brazil | KJ002419 | KJ002376 | This study |
| Ss340 | - | *Sporothrix brasiliensis* | Feline sporotrichosis | Guarulhos – SP, Brazil | KJ002420 | KJ002377 | This study |
| Ss341 | - | *Sporothrix brasiliensis* | Feline sporotrichosis | São Paulo – SP, Brazil | KJ002421 | KJ002378 | This study |
| Ss342 | - | *Sporothrix brasiliensis* | Feline sporotrichosis | São Paulo – SP, Brazil | KJ002422 | **-** | This study |
| Ss343 | - | *Sporothrix brasiliensis* | Feline sporotrichosis | São Paulo – SP, Brazil | KJ002423 | KJ002379 | This study |
| Ss344 | - | *Sporothrix brasiliensis* | Feline sporotrichosis | Diadema – SP, Brazil | KJ002424 | **-** | This study |
| Ss345 | - | *Sporothrix brasiliensis* | Feline sporotrichosis | Guarulhos – SP, Brazil | KJ002425 | KJ002380 | This study |
| Ss346 | - | *Sporothrix brasiliensis* | Feline sporotrichosis | São Paulo – SP, Brazil | KJ002426 | **-** | This study |
| Ss347 | - | *Sporothrix brasiliensis* | Feline sporotrichosis | São Paulo – SP, Brazil | KJ002427 | KJ002381 | This study |
| Ss349 | - | *Sporothrix brasiliensis* | Feline sporotrichosis | São Paulo – SP, Brazil | KJ002428 | KJ002382 | This study |
| Ss350 | - | *Sporothrix brasiliensis* | Feline sporotrichosis | São Paulo – SP, Brazil | KJ002429 | KJ002383 | This study |
| Ss351 | - | *Sporothrix brasiliensis* | Feline sporotrichosis | São Paulo – SP, Brazil | KJ002430 | **-** | This study |
| Ss352 | - | *Sporothrix brasiliensis* | Feline sporotrichosis | São Paulo – SP, Brazil | KJ002431 | KJ002384 | This study |
| Ss353 | - | *Sporothrix brasiliensis* | Feline sporotrichosis | São Paulo – SP, Brazil | KJ002432 | KJ002385 | This study |
| Ss354 | - | *Sporothrix brasiliensis* | Feline sporotrichosis | São Paulo – SP, Brazil | KJ002433 | KJ002386 | This study |
| Ss355 | - | *Sporothrix brasiliensis* | Feline sporotrichosis | São Paulo – SP, Brazil | KJ002434 | KJ002387 | This study |
| Ss356 | - | *Sporothrix brasiliensis* | Feline sporotrichosis | São Paulo – SP, Brazil | KJ002435 | KJ002388 | This study |
| Ss357 | - | *Sporothrix brasiliensis* | Feline sporotrichosis | São Paulo – SP, Brazil | KJ002436 | KJ002389 | This study |
| Ss358 | - | *Sporothrix brasiliensis* | Feline sporotrichosis | São Paulo – SP, Brazil | KJ002437 | KJ002390 | This study |
| **Isolate code** | **CBS Number** | **Species** | **Source** | **Origin** | **Genbank code** | | **References** |
|  |  |  |  |  | ***CAL*^a^** | ***EF1-α*^b^** |  |
| Ss359 | - | *Sporothrix brasiliensis* | Feline sporotrichosis | São Paulo – SP, Brazil | KJ002438 | **-** | This study |
| Ss360 | - | *Sporothrix brasiliensis* | Feline sporotrichosis | São Paulo – SP, Brazil | KJ002439 | KJ002391 | This study |
| Ss361 | - | *Sporothrix brasiliensis* | Feline sporotrichosis | São Paulo – SP, Brazil | KJ002440 | KJ002392 | This study |
| Ss362 | - | *Sporothrix brasiliensis* | Feline sporotrichosis | São Paulo – SP, Brazil | KJ002441 | KJ002393 | This study |
| Ss06 | CBS 132922 | *Sporothrix globosa* | Human sporotrichosis | Belo Horizonte – MG, Brazil | JF811336 | KC576545 | [1-3] |
| Ss41 | CBS 132923 | *Sporothrix globosa* | Human sporotrichosis | Fortaleza – CE, Brazil | JF811337 | KC576565 | [1-3] |
| Ss49 | CBS 132924 | *Sporothrix globosa* | Human sporotrichosis | Goiânia – GO, Brazil | JF811338 | KC576566 | [1-3] |
| CBS 120340 ^T^ | CBS 120340 | *Sporothrix globosa* | Human sporotrichosis | Spain | AM116908 | KC576608 | [4, 5] |
| FMR 8598 | CBS 130116 | *Sporothrix globosa* | Human sporotrichosis | Spain | AM116903 | KC576638 | [4, 5] |
| FMR 8595 | CBS 130104 | *Sporothrix globosa* | Human sporotrichosis | Spain | AM116905 | KC576609 | [4, 5] |
| ATCC 18616 ^T^ | CBS 937.72 | *Sporothrix luriei* | Human sporotrichosis | South Africa | AM747302 | KC576615 | [7] |
| Ss132 | CBS 132927 | *Sporothrix mexicana* | Human sporotrichosis | São Paulo – SP, Brazil | JF811340 | KC576590 | [1-3] |
| Ss133 | CBS 132928 | *Sporothrix mexicana* | Human sporotrichosis | Recife – PE, Brazil | JF811341 | KC576591 | [1-3] |
| CBS 120341 ^T^ | CBS 120341 | *Sporothrix mexicana* | Environmental, soil rose tree | Mexico | AM398393 | KC576611 | [5] |
| CBS 120342 | CBS 120342 | *Sporothrix mexicana* | Environmental, carnation leaves | Mexico | AM398392 | KC576610 | [5] |
| CBS 302.73 ^T^ | CBS 302.73 | *Sporothrix pallida* | Environmental, soil | United Kingdom | AM398396 | KC576612 | [5] |
| CBS 111110 | CBS 111110 | *Sporothrix pallida* | *Zootermopsis nevadensis* | Germany | AM398382 | KC576613 | [5] |
| HOL3 | - | *Sporothrix pallida* | Environmental, soil | Holland | HQ686040 | **-** | [8] |
| BG6 | - | *Sporothrix pallida* | Environmental, soil | Spain | HQ692915 | **-** | [8] |
| CBS 938.72 | CBS 938.72 | *Sporothrix schenckii* | Human sporotrichosis | France | AM490340 | KC576637 | [5] |
| **Isolate code** | **CBS Number** | **Species** | **Source** | **Origin** | **Genbank code** | | **References** |
|  |  |  |  |  | ***CAL*^a^** | ***EF1-α*^b^** |  |
| FMR 8679 | - | *Sporothrix schenckii* | Human sporotrichosis | Argentina | AM117445 | **-** | [4, 5] |
| FMR 8607 | - | *Sporothrix schenckii* | Human sporotrichosis | Peru | AM117428 | **-** | [4, 5] |
| FMR8608 | - | *Sporothrix schenckii* | Human sporotrichosis | Peru | AM117441 | **-** | [4, 5] |
| IHEM 3787 | - | *Sporothrix schenckii* | NK | South Africa | AM117435 | **-** | [4, 5] |
| IHEM 15486 | - | *Sporothrix schenckii* | Human sporotrichosis | Peru | AM117432 | **-** | [4, 5] |
| IHEM 3774 | - | *Sporothrix schenckii* | Human sporotrichosis | Colombia | AM117447 | **-** | [4, 5] |
| UTHSC 05-2843 | - | *Sporothrix schenckii* | Human sporotrichosis | USA | AM399012 | **-** | [5] |
| UTHSC 05-802 | - | *Sporothrix schenckii* | Human sporotrichosis | USA | AM399008 | **-** | [5] |
| Ss01 | CBS 132961 | *Sporothrix schenckii* | Feline sporotrichosis | São Paulo – SP, Brazil | KC693828 | KC576540 | [2, 3] |
| Ss02 | CBS 132962 | *Sporothrix schenckii* | Human sporotrichosis | Porto Alegre – RS, Brazil | KC693829 | KC576541 | [2, 3] |
| Ss03 | CBS 132963 | *Sporothrix schenckii* | Human sporotrichosis | Porto Alegre – RS, Brazil | JX077117 | KC576542 | [1-3] |
| Ss26 | CBS 132965 | *Sporothrix schenckii* | Human sporotrichosis | Curitiba – PR, Brazil | KC693841 | KC576557 | [2, 3] |
| Ss63 | CBS 132968 | *Sporothrix schenckii* | Human sporotrichosis | Vila Velha – ES, Brazil | JX077123 | KC576573 | [1-3] |
| Ss80 | CBS 132969 | *Sporothrix schenckii* | Human sporotrichosis | Rio de Janeiro – RJ, Brazil | JX077125 | KC576583 | [1-3] |
| Ss111 | CBS 132971 | *Sporothrix schenckii* | Human sporotrichosis | São Paulo – SP, Brazil | KC693860 | KC576587 | [2, 3] |
| CBS 359.36 ^T^ | CBS 359.36 | *Sporothrix schenckii* | Human sporotrichosis | USA | AM117437 | KC576614 | [4, 5] |
| CBS 141.36 ^T^ | CBS 141.36 | *Grosmannia serpens* | Environmental, wood | Italy | JN135300 | **-** | [9] |

^a^Calmodulin (*CAL*) data set, amplified and sequenced using the primers CL1 and CL2A [10].

^b^Translation Elongation Factor (*EF1-α*) dataset, based on amplification using the primers EF1-F and EF1-R [2].

IPEC, Instituto de Pesquisa Clínica Evandro Chagas, Fiocruz, Brazil; FMR, Facultat de Medicina i Ciències de la Salut, Reus, Spain; CBS, Centraalbureau voor Schimmelcultures, Utrecht, The Netherlands; UTHSC, Fungus Testing Laboratory, University of Texas Health Science Center; IHEM, BCCM/IHEM Biomedical Fungi and Yeasts Collection, Belgium; ATCC: American Type Culture Collection, Manassas, USA; NK, not known; T, type strain; All “Ss” strains belong to the culture collection of Federal University of São Paulo (UNIFESP).

**References**

1. Rodrigues AM, de Hoog S, de Camargo ZP: **Emergence of pathogenicity in the *Sporothrix schenckii* complex**. *Med Mycol* 2013, **51**:405-412.

2. Rodrigues AM, de Melo Teixeira M, de Hoog GS, Schubach TMP, Pereira SA, Fernandes GF, Bezerra LML, Felipe MS, de Camargo ZP: **Phylogenetic analysis reveals a high prevalence of *Sporothrix brasiliensis* in feline sporotrichosis outbreaks**. *PLoS Negl Trop Dis* 2013, **7**:e2281.

3. Rodrigues AM, de Hoog GS, Zhang Y, Camargo ZP: **Emerging sporotrichosis is driven by clonal and recombinant *Sporothrix* species**. *Emerg Microbes Infect* 2014, **3**:e32.

4. Marimon R, Gené J, Cano J, Trilles L, Dos Santos Lazéra M, Guarro J: **Molecular phylogeny of *Sporothrix schenckii***. *J Clin Microbiol* 2006, **44**:3251-3256.

5. Marimon R, Cano J, Gené J, Sutton DA, Kawasaki M, Guarro J: ***Sporothrix brasiliensis*, *S. globosa*, and *S. mexicana*, three new *Sporothrix* species of clinical interest**. *J Clin Microbiol* 2007, **45**:3198-3206.

6. Silva-Vergara ML, de Camargo ZP, Silva PF, Abdalla MR, Sgarbieri RN, Rodrigues AM, dos Santos KC, Barata CH, Ferreira-Paim K: **Disseminated *Sporothrix brasiliensis* infection with endocardial and ocular involvement in an HIV-infected patient**. *Am J Trop Med Hyg* 2012, **86**:477-480.

7. Marimon R, Gené J, Cano J, Guarro J: ***Sporothrix luriei*: a rare fungus from clinical origin**. *Med Mycol* 2008, **46**:621-625.

8. Romeo O, Scordino F, Criseo G: **New insight into molecular phylogeny and epidemiology of *Sporothrix schenckii* species complex based on calmodulin-encoding gene analysis of Italian isolates**. *Mycopathologia* 2011, **172**:179-186.

9. Duong TA, de Beer ZW, Wingfield BD, Wingfield MJ: **Phylogeny and taxonomy of species in the *Grosmannia serpens* complex**. *Mycologia* 2012, **104**:715-732.

10. O’Donnell K, Nirenberg H, Aoki T, Cigelnik E: **A multigene phylogeny of the *Gibberella fujikuroi* species complex: Detection of additional phylogenetically distinct species**. *Mycoscience* 2000, **41**:61-78.
