# Supplementary material for: Feline sporotrichosis due to Sporothrix brasiliensis: an emerging animal infection in São Paulo, Brazil
Source: BMC Vet Res. 2014 Nov 19;10:269. doi: 10.1186/s12917-014-0269-5 (PMC4244058; doi:10.1186/s12917-014-0269-5)
Supplement: Additional file 2: — Median-joining haplotype network of Sporothrix schenckii complex isolates, comparing all EF1-α haplotypes described in the ongoing epidemics in Rio de Janeiro, Rio Grande do Sul, and São Paulo. Isolates recovered in the São Paulo epidemics (2011-2013) share the same haplotype (H9) as previous outbreaks in Rio de Janeiro (1998-2012) reported by Rodrigues et al. [4]. The size of the circumference is proportional to the haplotype frequency. Isolates are coded, and their frequencies are represented by geographic region of isolation. Black dots (median vectors) represent unsampled or extinct haplotypes in the population. [file 12917_2014_269_MOESM2_ESM.doc]

**Additional file 2 Median-joining haplotype network of *Sporothrix schenckii* complex isolates, comparing all *EF1-α* haplotypes described in the ongoing epidemics in Rio de Janeiro, Rio Grande do Sul, and São Paulo.** Isolates recovered in the São Paulo epidemics (2011-2013) share the same haplotype (H9) as previous outbreaks in Rio de Janeiro (1998-2012) reported by Rodrigues *et al*. (2013) [1]. The size of the circumference is proportional to the haplotype frequency. Isolates are coded, and their frequencies are represented by geographic region of isolation. Black dots (median vectors) represent unsampled or extinct haplotypes in the population.


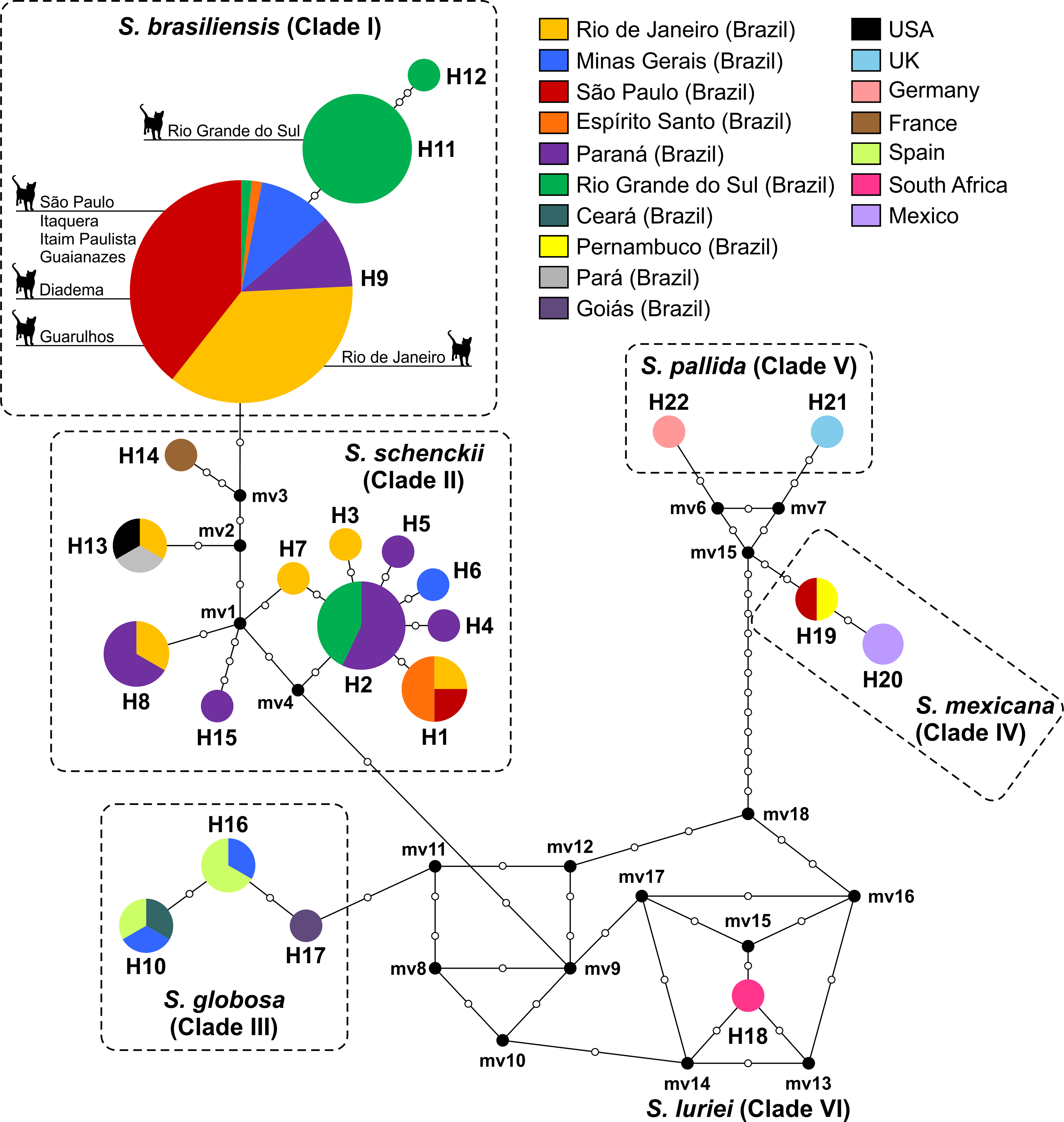


**Reference**

1. Rodrigues AM, de Melo Teixeira M, de Hoog GS, Schubach TMP, Pereira SA, Fernandes GF, Bezerra LML, Felipe MS, de Camargo ZP: **Phylogenetic analysis reveals a high prevalence of *Sporothrix brasiliensis* in feline sporotrichosis outbreaks**. *PLoS Negl Trop Dis* 2013, **7**:e2281.
